# Discovery of carotenoid red-shift in endolithic cyanobacteria from the Atacama Desert

**DOI:** 10.1038/s41598-017-11581-7

**Published:** 2017-09-11

**Authors:** Petr Vítek, Carmen Ascaso, Octavio Artieda, María Cristina Casero, Jacek Wierzchos

**Affiliations:** 1 Global Change Research Institute CAS, Bělidla 986/4a, 603 00 Brno, Czech Republic; 2 Museo Nacional de Ciencias Naturales, CSIC, c/ Serrano 115 dpdo, 28006 Madrid, Spain; 3 Departamento Biología Vegetal, Ecología y Ciencias de la Tierra, and IACYS, Universidad de Extremadura, 10600 Plasencia, Spain

## Abstract

The biochemical responses of rock-inhabiting cyanobacteria towards native environmental stresses were observed *in vivo* in one of the Earth’s most challenging extreme climatic environments. The cryptoendolithic cyanobacterial colonization, dominated by *Chroococcidiopsis* sp., was studied in an ignimbrite at a high altitude volcanic area in the Atacama Desert, Chile. Change in the carotenoid composition (red-shift) within a transect through the cyanobacteria dominant microbial community (average thickness ~1 mm) was unambiguously revealed in their natural endolithic microhabitat. The amount of red shifted carotenoid, observed for the first time in a natural microbial ecosystem, is depth dependent, and increased with increasing proximity to the rock surface, as proven by resonance Raman imaging and point resonance Raman profiling. It is attributed to a light-dependent change in carotenoid conjugation, associated with the light-adaptation strategy of cyanobacteria. A hypothesis is proposed for the possible role of an orange carotenoid protein (OCP) mediated non-photochemical quenching (NPQ) mechanism that influences the observed spectral behavior. Simultaneously, information about the distribution of scytonemin and phycobiliproteins was obtained. Scytonemin was detected in the uppermost cyanobacteria aggregates. A reverse signal intensity gradient of phycobiliproteins was registered, increasing with deeper positions as a response of the cyanobacterial light harvesting complex to low-light conditions.

## Introduction

Endolithic (rock-inhabiting) phototrophic microorganisms, dominated by *Chroococcidiopsis* sp., were first described from the Negev Desert, Israel in the pioneering research study by Imre E. Friedmann and Roseli Ocampo-Friedmann^1^. This work was followed by the first description of endolithic cyanobacteria from orthoquartzite in one of the Earth’s most extreme climatic environments in the Antarctic Dry Valleys^2^. The first Raman spectroscopic identifications of biomolecules associated with endolithic phototrophs from the sandstones of the McMurdo Dry Valleys were described later^3, 4^.

The Atacama Desert represents another extreme desert environment, and as one of the driest places on Earth provides a natural laboratory to study life ‘at the edge’. Porous and semi-translucent rocks of various origins were found to provide a refuge for photosynthetic microorganisms that have to cope with multiple synergistic stress factors^5, 6^ comprised of extremes of aridity and solar irradiation. The diverse rock substrates are comprised of evaporites - halite^5, 7, 8^, gypsum^6, 9, 10^, calcite^11^; as well as volcanic rocks such as ignimbrite^12^.

Photosynthetic organisms have to balance between sufficient photosynthetically active radiation (PAR) as an energy source, and an excess of light, which may result in the harmful production of reactive oxygen species. Carotenoids are a group of biomolecules that play an important role in this regard. Carotenoid pigments are involved in photosynthesis, photo-protection, and membrane stabilization. Carotenoid molecules act as protective agents because of their ability to prevent the formation of singlet oxygen (^1^O_2_) by rapidly quenching chlorophyll triplet states, as well as having the potential for rapid direct scavenging of singlet oxygen^13–16^.

It is well known that carotenoids have an important photo-protective role through dissipation of excess excitation energy in the xanthophyll cycle, which is considered a key photoprotective mechanism in higher plants and algae^17, 18^. Cyanobacteria also protect themselves against light-induced stress, caused by an excess of absorbed energy exceeding the rate of carbon fixation. The photo-protective mechanism of carotenoids in cyanobacteria works in a completely different manner than in higher plants and algae. It is based on the interaction between the carotenoid protein with phycobilisome. The Orange Carotenoid Protein (OCP) was first described by Holt and Krogman^19^, and the structure was determined by Kerfeld *et al*.^20^. However, its function was only described in the past decade in 2006^21^. This water-soluble protein is responsible for an increase in heat dissipation induced by intense blue-green light radiation (not by the orange to red wavelengths)^21, 22^. It contains N-terminal and C-terminal domains with a single non-covalently bound keto-carotenoid molecule (echinenone or hydroxyechinenone) spanning both domains^23^.

A characteristic feature of the structure of carotenoid resides in the long conjugated double-bond system, composed of isoprenoic units, which results in strong Raman scattering. Carotenoids have two strong Raman bands in the 1490–1540 and 1150–1160 cm^−1^ regions, due to in-phase ν_1_(C=C) and ν_2_(C–C) stretching vibrations of the polyene chain, respectively. A feature of medium intensity occurs around 1005 cm^−1^, corresponding to the in-plane rocking modes of the CH_3_ groups attached to the polyene chain^24, 25^. Wilson *et al*.^26^ documented that the red form of the OCP (OCP^R^) is accompanied by a change in π-conjugation of the carotenoid polyene chain. Withnall *et al*.^27^ described how the conjugation of the polyene chain particularly affected the position of the ν_1_(C=C) band. In accordance with this, the Raman shift of the ν_1_(C=C) toward a lower wavenumber position in the case of OCP^R^ is observed, as has been described by Wilson *et al*.^26^ and others. Simultaneously, FTIR spectra revealed H-bond loosening, reflected in the carbonyl region of the spectra. The Raman spectroscopic data were confirmed by Leverenz *et al*.^28^ who also described the differences in the weak to medium intensity bands. The configuration of the carotenoid, later revealed by Leverenz *et al*.^29^, showed an increased planarity of the polyene and reduced β-ring torsions in the red carotenoid protein, consistent with the Raman spectroscopic data. Detailed structural changes associated with activation of the OCP were described by Gupta *et al*.^30^.

Here, for the first time, we have demonstrated evidence of the carotenoid red shift attributed to photo-protection is occurring both *in situ* and *in vivo* in natural endolithic microbial ecosystems (primarily composed of *Chroococcidiopsis* sp. cyanobacteria) within a rock micro-habitat, provided by a porous ignimbrite of volcanic origin from the hyperarid region of the Atacama Desert.

## Results

### Climatic context of the area

The mean annual rainfall of the hyperarid core of the Atacama Desert was reported to be less than 1 mm yr^−1^ ^31^. The mean atmospheric relative humidity described from four hyperarid sites in the desert varied from 17.3% to 28.8%^32^.

The sampling zone lies close to Cordon de Lila and 9 km north of the Lomas de Tilocalar area, from where the cryptoendolithic colonization of ignimbrite rocks was recently described^12, 33, 34^. The extreme aridness of this area, expressed by an aridity index (AI) reported to be about 0.0075^12^, and with annual total rainfall between 22 and 27 mm^6^. A detailed study of the long-term environmental parameters, such as air temperature (T), air relative humidity (RH), and photosynthetic active radiation (PAR) was recently reported for the Cordon de Lila area^6^. These data indicate an extremely low value of a mean annual RH of 16.5%, and mean maximum daily PAR values up to 2554 μmol photons m^−2^ s^−1^ have been recorded. According to the irradiance map presented in Rondanelli *et al*.^35^, the solar irradiance in this region is some of the highest on Earth. Moreover, a high level of UV radiation was reported by Cabrol *et al*.^36^ for the cloudless high-altitude Andean regions, with very low RH values, a low-ozone column, and a UV index of 43.3. To summarize, this location is characterized by extreme solar irradiation as well as extreme aridity.

### Ignimbrite microbial endolithic habitat

**Figure 1:**
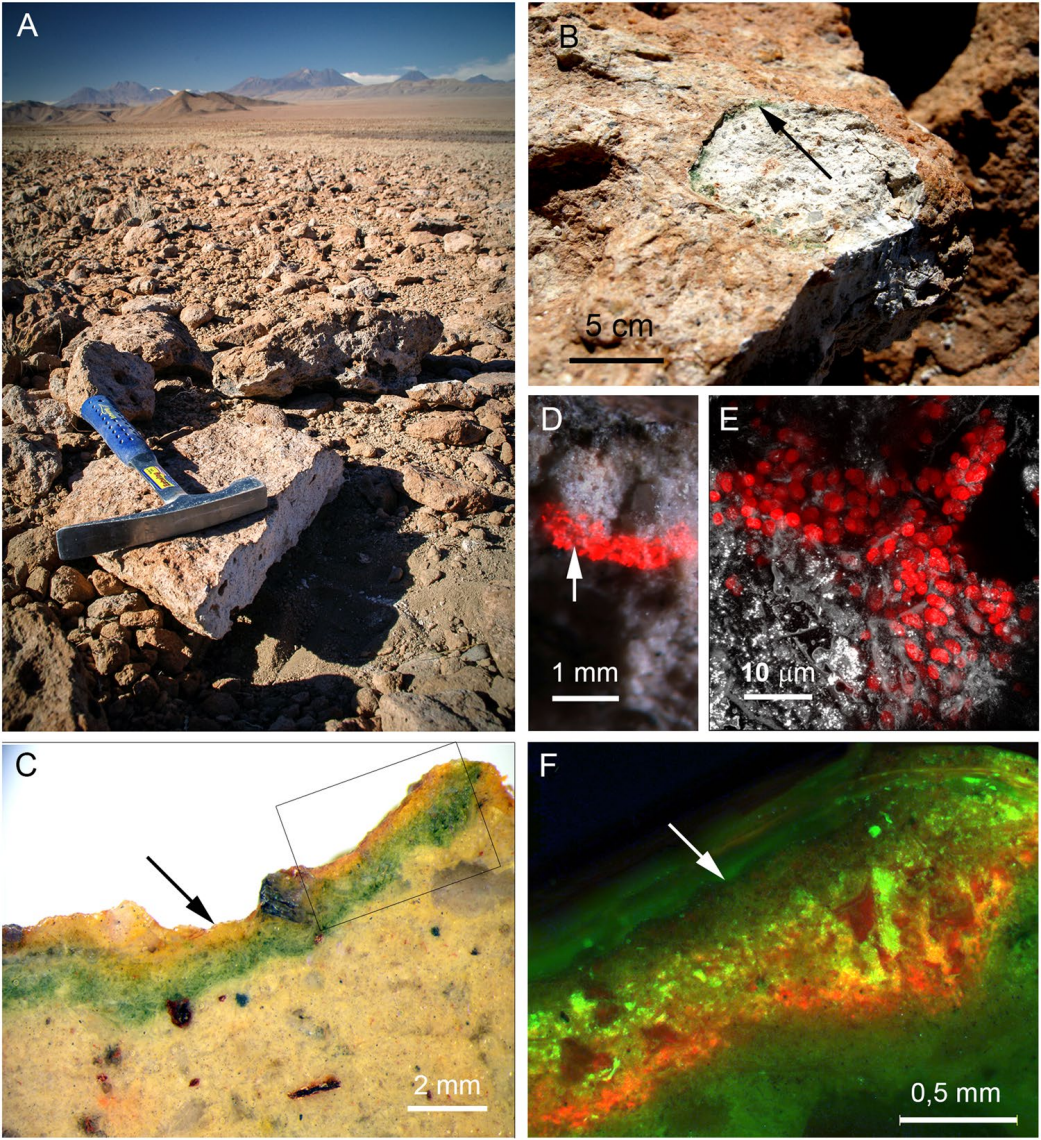
Sampling location, studied ignimbrite and fluorescemce microscopy of the endolithic colonization layer. Landscape of the Atacama Desert at the sampling location (**A**). Ignimbrite rock with hammer detached rock surface containing the endolithic microbial communities, indicated by arrow (**B**). Transversal section through the ignimbrite (incident light) shows a distinct endolithic green colour layer beneath the rock surface (arrow) (**C**). CLSM image (**D**) and higher magnification image (**E**) shows endolithic autofluorescent (red signal) cyanobacteria pointed in (**D**) by a white arrow; white signal in CLSM image (**E**) originated by mineral particles reflecting laser light. Rectangle marked area in (**C**) is shown in fluorescent microscopy mode in the image (**F**). Note, white arrow points to the rock surface; the green signal reveals the position of the heterotrophic bacteria, and the red signal shows the position of the autofluorescent cyanobacteria.

Many of the ignimbrite rocks in the sampling area showed endolithic colonization, which was visible as a narrow 1–2 mm green layer beneath the rock surface (Fig. 1B). This colonization was dominated by phototrophic microorganisms, cyanobacteria, with a strong autofluorescence signal when visualized by Confocal Laser Scanning Microscopy CLSM (Fig. 1D,E). Note that the cyanobacteria cells were forming compact aggregates filing out the pore spaces among the mineral particles (Fig. 1E). The colonization layer ran parallel to the ignimbrite surface despite surface roughness (Fig. 1C). Phylogenetic analyses of these ignimbrite endolithic microbial communities reveals that a cyanobacterium belonging to *Chroococcidiopsis* sp. is the dominant phototrophic microorganism, with a relative abundance at the phylum level of 82–85%. The remaining 15–17% belongs to the Chloroflexi, Actinobacteria, and Proteobacteria taxa^34^.

**Figure 2:**
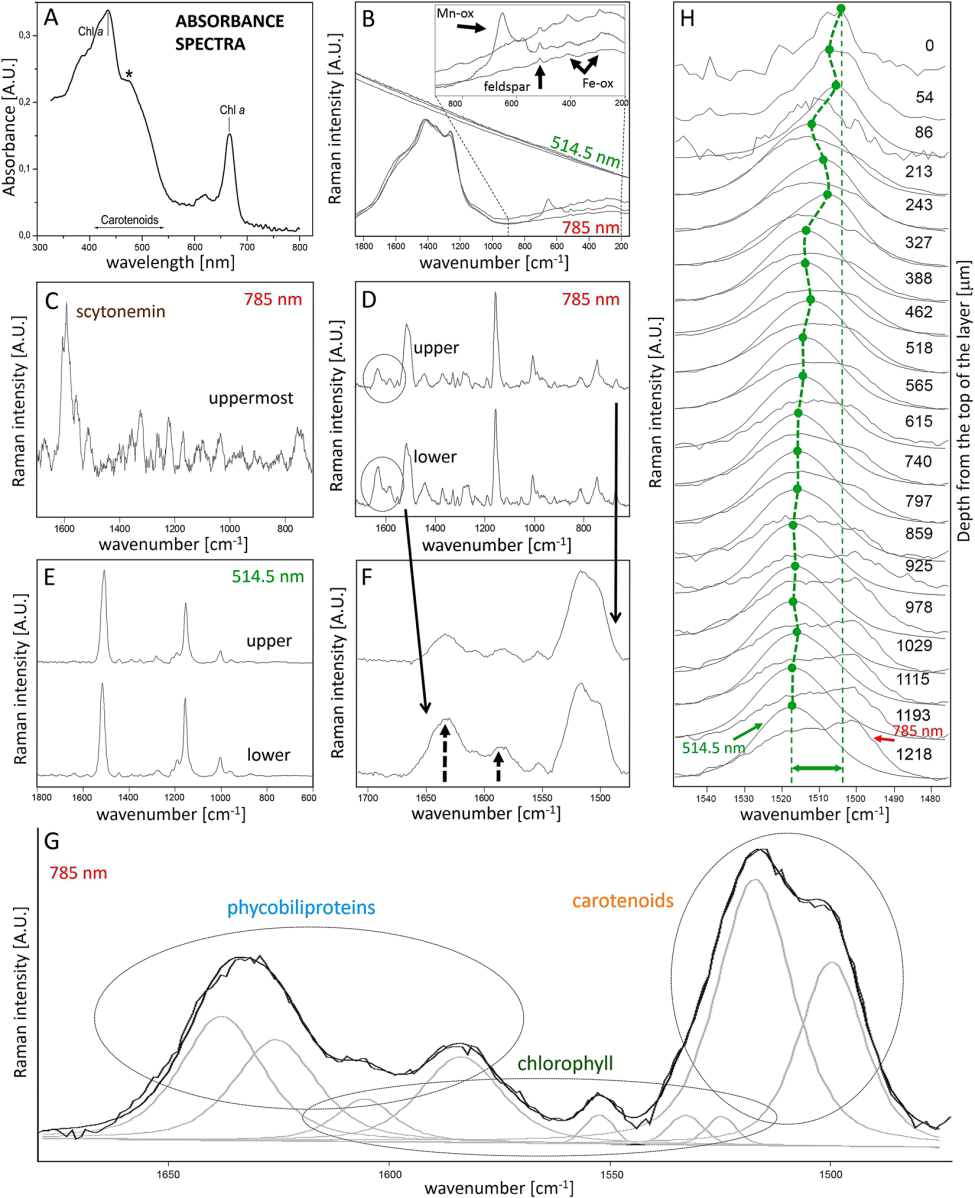
Raman spectroscopic features and absorbance spectra of the cyanobacterial pigments. Absorbance spectrum of acetone-extracted pigments in the range of 320–800 nm; asterisk points to the carotenoid induced shoulder (**A**). Typical spectral record from the ignimbrite mineral matrix as obtained by 785 nm and 514.5 nm laser wavelengths (**B**). Scytonemin features were detected in some areas of the uppermost parts of the colonized zone (**C**). Raman bands using 785 nm (red) excitation wavelength obtained at the upper and lower positions of the colonized zone – average of five spectra from each zone (**D**), with enhanced relative content of phycobiliproteins indicated by arrows (**F**). Spectral record from the upper and lower positions obtained by green (514.5 nm) laser excitation is presented in (**E**). In (**G**), the curve-fitting of the spectral record between 1480–1680 cm^−1^ shows individual bands corresponding to carotenoids, chlorophyll *a*, and phycobiliproteins. In (**H**), the depth profile (corresponding to profile 3 in Fig. 3) is depicted comparing both green (514.5 nm) and red (785 nm) laser excitation applied to identical cell aggregates.

Fluorescence microscopy imaging (Fig. 1F) reveals that cyanobacteria (red autofluorescence) are occupying a lower position within the endolithic colonization layer, and that heterotrophic bacteria (green autofluorescence) appear closer to the rock surface. The Raman spectra obtained by 785 nm excitation show those bands corresponding to three major groups of cyanobacterial pigments - carotenoids, phycobiliproteins, and chlorophyll (Fig. 2). The Raman features of scytonemin were detected in some cyanobacterial cell aggregates in the upper position of the colonized layer; although the observation of this UV-screening pigment was not common within the colonized zones studied.

The absorbance spectrum of the pigment extracts obtained from the endolithic colonization zone is shown in Fig. 2A. This extract showed high absorbance values for chlorophyll *a*, characteristic for cyanobacteria pigment. However, the shoulder at around 470 nm indicates the absorption value lies within the carotenoid absorbance range.

### Pigment distribution

**Figure 3:**
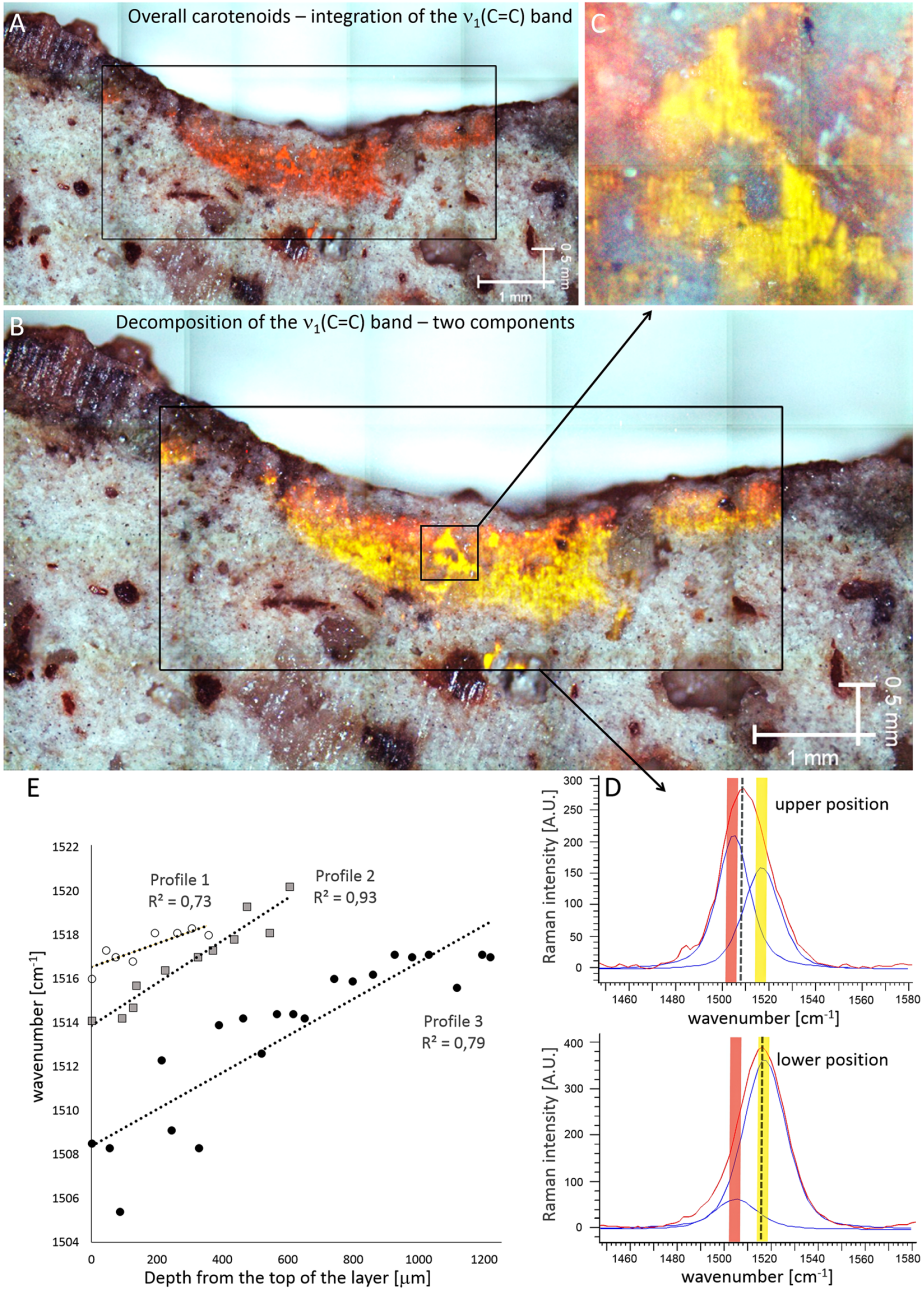
Raman imaging (in false color) shows distribution of the integrated ν(C=C) band. The overall carotenoid signal (**A**). Gradient in carotenoid composition with red-shifted carotenoid enhanced within the upper part of the colonized zone (**B**, detail in **C**). Examples of the curve-fitted ν(C=C) band from both positions (top, bottom) are shown in (**D**) Correlation of the carotenoid ν(C=C) band position with depth, obtained by manual point analysis from three different profiles, is presented in (**E**).

Employing green (514.5 nm) and red (785 nm) excitation sources for analysis of the cyanobacteria resulted in two different spectral records. Two clearly distinct Raman bands of carotenoids corresponding to ν_1_(C=C) vibration were observed by single point Raman analysis using 785 nm excitation at 1519 and 1502 cm^−1^. Due to the significant difference of the positions of these two bands, this was interpreted as a result of the presence of carotenoids with distinct numbers of conjugated double bonds (Fig. 2G). When employing a 514.5 nm laser line, one split band of the carotenoid ν_1_(C=C) appears, and shifts its position at different depths within the cyanobacterial zone (Fig. 2E,H). The different spectral appearance of the band at the two distinct excitation wavelengths is interpreted as a result of the selective resonance enhancement of a specific carotenoid component, which dominates the spectra when using a 514.5 nm laser. This is in accordance with the Raman spectra of cyanobacteria provided by Vítek *et al*.^37^. Raman bands corresponding to the surrounding mineral matrix occur especially in the wavenumber region below 1000 cm^−1^ (Fig. 2B). The band due to feldspar occur at 513 cm^−1^. Bands of weak intensity below 500 cm^−1^ are attributed to Fe-oxides, whereas the medium to strong band at 651 cm^−1^ with shoulder at 576 cm^−1^ is probably due to Mn-oxide that occur within dark-colored areas close to the surface. Hence, no overlap with the studied Raman signal of pigments occur. The fluorescence background (especially in the case of 785 nm) affected the spectra of the studied pigments, however it was effectively substracted (see Methods). Raman mapping employing a 514.5 nm laser line show no systematic change in the intensity of the overall carotenoid Raman signal when integrating the ν_1_(C=C) band (Fig. 3A). However, a clear gradient of the ν_1_(C=C) band composition was observed when curve-fitted for the two bands (Fig. 3B). The intensity of the lower wavenumber component is enhanced within the upper part of the transect through the colonized zone. This observation was confirmed by the detailed mapping of the smaller zone (Fig. 3C) using a higher magnification objective (20x). The band of higher wavenumber position is located at approx. 1519 cm^−1^, whereas the low wavenumber band is observed at 1502 cm^−1^. Examples of the curve-fitted bands extracted from the two distinct positions (upper and lower) are depicted in Fig. 3D.

The results obtained by the Raman imaging data were validated using a different analytical approach. A single point resonance Raman analysis was performed through the three different profiles of the cyanobacterial layer from different positions (Fig. 3E). A strong correlation of the ν_1_(C=C) carotenoid band position with depth was observed in all profiles, thus confirming the results described above (Fig. 3E).

Employing the 514.5 nm and 785 nm laser lines shows distinct spectral behaviors related to depth when comparing the two excitation wavelengths. The relative increase of the red shifted carotenoid band detected by 514.5 nm moving toward positions closer to the surface of the colony is not observed using the 785 nm wavelength (Fig. 2H). Both analyses were obtained from identical cell aggregates at a specific depth. The 785 nm excitation shows no enhancement of the red shifted band closer to the surface position; but instead possesses a more enhanced red-shifted band at lower positions. The uppermost two spectra in Fig. 2H possess a weaker resonance Raman signal of carotenoids superimposed on a stronger fluorescence background; and scytonemin Raman features dominate the spectral record when using 785 nm excitation, as shown in baseline-corrected spectra (Fig. 2C).

A strong signal due to phycobiliproteins in the 1560–1660 cm^−1^ range was detected at 785 nm excitation, arising from stretching vibrations of C=C in methane bridges and pyrrole rings, with some contribution from C=N stretchings^38^. Medium to weak bands of phycobiliproteins were detected at 1373, 1287, 1267, 1237, 1188, 1051, 1110, 817, and 667 cm^−1^. Depth-dependent variations of the relative signal intensity of phycobiliproteins were registered by point Raman measurements (Fig. 2D,F), and also confirmed by Raman imaging (unpublished data). The Raman intensity of the phycobiliproteins is enhanced at deeper positions from the rock surface.

The red excitation also provides corroborative chlorophyll features at 1556, 1329, 1189, 1146sh, 918, 745 cm^−1^, as well as other bands of weaker intensity. The chlorophyll signal intensity relative to overall carotenoid signal remains at comparable levels at different depths.

## Discussion

The exact position of the ν_1_(C=C) Raman band of carotenoids is clearly depth-dependent within the colonization zones studied. The phenomenon is confirmed by both Raman imaging and point Raman analysis. A strong correlation of the ν_1_(C=C) band position with depth, obtained from three different rock profiles, was observed. Offsets between the datasets from the different profiles is interpreted as the result of varying light conditions between the individual microhabitats. The longer-chained polyene chain within a carotenoid molecule is responsible for the ν_1_(C=C) wavenumber shift towards a lower value^25, 27^. As described by de Oliveira *et al*.^39^, the shift of the band is also affected by other factors, such as bonding to other molecules. A small change may result from a change in the molecular termination or isomerization^27, 39, 40^. In this study, a significant shift was observed in the same type of cyanobacteria. Moreover, curve-fitting of the imaging dataset is in accordance with the changing intensity ratio of the two spectral components with a significantly distinct wavenumber position. Hence, the observed gradient in carotenoid spectral shift is interpreted to reflect a change in the conjugation of the polyene chain. This is the most important outcome of the study.

The observation of two distinct ν_1_ bands is a common situation when analyzing endolithic cyanobacteria using 785 nm laser excitation. We can report a similar spectral pattern, obtained on the cyanobacterial colony inhabiting gypsum, from at least two distinct areas in the Atacama Desert [ref. 37, unpublished results]. Imaging of the gypsum-inhabiting algal colonization provided different results, showing a depth-dependent change in the carotenoid ν_1_ band intensity^41^. The carotenoid content based on the ν_1_ band intensity increased towards the surface, and is interpreted as a photo-protection strategy. However, no shift in the ν_1_ band position with depth was observed in that study within the imaging dataset.

Phycobiliproteins were detected in all positions through the depth profile, except in the uppermost parts rich in scytonemin where a huge fluorescent background resulted in poor Raman spectra. The Raman features of phycobiliproteins were enhanced with deeper positions. The light-dependent biosynthesis of phycobiliproteins had been spectroscopically observed previously by Villar *et al*.^42^, who examined the Raman spectroscopic features of phycocyanin in cyanobacteria inhabiting volcanic rocks in Svalbard (Norway). This finding was interpreted as the response of cyanobacteria to low-light conditions in deeper parts, enhancing the light harvesting capability. The more intense red autofluorescence at the lower parts of the colonized zone are in accordance with the higher content of phycobiliproteins detected by Raman spectroscopy.

Stratification of pigment composition within gypsum-inhabiting cyanobacteria was described by Oren *et al*.^43^ from a wet hypersaline environment from the solar salterns in Eilat (Israel). Using HPLC, a specific mixture of various carotenoids was observed within individual layers (differing in their cyanobacterial genera), and were interpreted as being dependent on the light conditions^43^. The Raman shifts of the ν_1_(C=C) carotenoid band positions obtained on these colonized layers of different coloration (orange, green, red) were documented by Culka *et al*.^44^. However, the individual layers are occupied by a distinct genus of cyanobacteria in the solar salterns, and the character of the ecosystem studied here is different. Here, a continuous evolution of the split ν_1_(C=C) band position with depth was observed within the same morphospecies of cyanobacteria as confirmed by microscopic observation composed by *Chroococcidiopsis* sp., as was demonstrated in phylogenetic studies^12, 33, 34^.

The observed carotenoid shift is interpreted as a light-dependent phenomenon. One of the protective functions of carotenoids is the rapid direct scavenging of singlet oxygen - if any is formed during photosynthesis or due to UV-irradiation. Otherwise, singlet oxygen would destroy the biomolecules that form the photosynthetic apparatus. The longer-chained carotenoids are able to cope more effectively with these toxic reactive oxygen species^45^. Therefore, one of the possible explanations for our observations is the adaptation of cyanobacteria to excessively harsh radiation through the synthesis of carotenoids that are more effective antioxidants, by their direct quenching of singlet oxygen.

Secondly, non-photochemical quenching (NPQ) may play an important role in the spectroscopic observations described. The OCP-based NPQ mechanism is activated by blue-green light, which induces conversion of the OCP into the red form (OCP^R^), thereby allowing for the interaction with one of the basal allophycocyanin cylinders within the core of phycobilisome^46, 47^. Hence, the OCP acts as a light sensor, signal propagator, and energy quencher^48^. Moreover, OCP can directly quench singlet oxygen as described by Krieger-Liszkay^49^. Boulay *et al*.^50^ suggested that the OCP-based photoprotective mechanism is widespread in cyanobacteria. Genes encoding for full-length OCP have been discovered in 90 out of 127 of the surveyed cyanobacterial genomes, including *Chroococcidiopsis* sp.^48^. Although tremendous progress in an understanding of cyanobacterial photoprotection has been achieved since 2003 (when the structure of OCP was determined^20^), the precise process involved in its function still remains to be elucidated (see recent review by Kirilovsky and Kerfeld^23^).

It is assumed that the laser excitation used for the resonance Raman analysis could initiate the OCP - OCP^R^ transition^51^. Authors studying isolated OCP and OCP^R^ have used various approaches to avoid laser-induced activation of OCP during analysis, in order to characterize both forms using Raman spectroscopy. These comprise rapid-flow conditions to minimize photoconversion in the registered volume of the sample^28, 51^, or the use of low temperature (10 K) under which the sample was maintained during the analysis^26^. On an isolated OCP, it has been demonstrated that the transition of OCP to OCP^R^ is reflected by a Raman shift of the ν_1_(C=C) towards lower positions, according to the change in conjugation^26, 28, 51^.

In our study, due to the thin colonization zone, it was not possible to separate and perform pigment extractions of the zones with a distinct carotenoid signal from the rock. For this purpose, *in-situ* and *in-vivo* micro-imaging techniques are excellent tools, maintaining the micro-scale context of the studied colonization in its original habitat. Dealing with the natural endolithic cyanobacterial community inside the rock habitat, we did not perform any of above described treatments in order to avoid possible laser-induced OCP to OCP^R^ photoconversion. We assumed that the green 514.5 nm line of the excitation laser would affect the OCP photoconversion, and that this would probably occur immediately, as mentioned by Maksimov *et al*.^51^. If this concept is true, the registered transition was more abundant within the upper parts of the colonized zone. The lack of an OCP contribution to the registered signal (whatever the position is) is supported by the weak bands around 983 cm^−1^, and the weak to medium spectral features in the region 1170–1400 cm^−1^ ^51^. The band at 983 cm^−1^ reflects out-of-plane distortions of the polyene chain (enhanced in OCP), and its intensity is low for a planar polyene like in OCP^R^ ^28^. In the 1170–1400 cm^−1^ region, two distinct peaks at 1186 and 1213 cm^−1^ should occur in the OCP spectra. These are replaced by the band around 1195 cm^−1^ in the spectra obtained, which is in accordance with the red form (OCP^R^)^28^ and consistent with an *all-trans* C40 carotenoid^28, 52^. Further work will have to follow to prove the role of the OCP-OCP^R^ transition in the observed spectral behavior. The interaction of green excitation light with carotenoids is supported by the noticeable difference in spectral behavior with depth between green (514.5 nm) and red (785 nm) excitation. Employing the red laser line does not lead to an enhancement of the low-wavenumber component with proximity to the surface.

## Concluding remarks

- Systematic depth-dependent Raman shift of the carotenoid ν_1_(C=C) band was observed at many transects of the sub-surface endolithic cyanobacterial layer within ignimbrite. This was confirmed by two different approaches: Raman mapping, and manual Raman profiling using point analysis (both in resonance conditions employing 514.5 nm excitation).
- This was interpreted as the need by cyanobacteria for more photo-protection with their proximity to the rock surface, where the cyanobacteria have to cope with an excess of light. The red-shifted carotenoids, with more conjugated double bonds in the polyenic chain, are supposed to be more effective in the direct quenching of singlet oxygen, or playing a role in the non-photochemical quenching (NPQ) mechanism of cyanobacteria.
- We propose the hypothesis that the more frequent OCP-OCP^R^ transition with proximity to the surface is the likely mechanism that influences the observed Raman shift. This is consistent with the previous point. The most significant result supporting this hypothesis is provided by the comparison of the two excitation wavelengths employed during Raman profiling – the green (514.5 nm) that resulted in the observed carotenoid red-shift of the ν_1_(C=C) band, and the red (785 nm) that did not. This is consistent with the OCP-OCP^R^ transition induced by blue-green light described in the literature.

## Methods

### Samples

The sampling site lies within the hyperarid zone of the Atacama Desert, in northern Chile; in the southern part of the Salar de Atacama basin (23°52′S; 069°8′W and 2900 m a.s.l.) in the large north-south trending Cordon de Lila range. This area is primarily covered by volcanic ash materials, with different sizes of ignimbrite boulders and smaller rocks (Fig. 1A) that originated during the Pliocene^53^. Frequently, the endolithic habitat, in the form of a narrow green layer just beneath the ignimbrite surface, can be clearly distinguished in freshly fractured substrate (Fig. 1B). Samples of ignimbrite with distinct signs of endolithic colonization were collected into sterile Whirl-Pack® bags, sealed, and stored in dry conditions at room temperature until analyses.

### Confocal and Fluorescent microscopy

Small fragments of ignimbrite, showing distinct signs of endolithic colonization as a green-colored layer beneath the rock surface, were moistened with distilled water, and the autofluorescence of the cyanobacteria cell aggregates were visualized *in situ* using a Leica TCS-SP5 confocal laser scanning microscope (CLSM) (Leica Microsystems Heidelberg GmbH, Mannheim, Germany). Red autofluorescence was viewed in the red channel (640 to 785 nm emission) using a 561 nm laser diode.

Other fragments of rocks were cut perpendicularly to the rock surface with a diamond saw, and this plane was stained with SYBR Green (Molecular Probes), a fluorochrome used for specific staining of bacterial cell nucleic acids (NA). Next, the endolithic microbial colonies were observed *in situ* using a Zeiss AxioImager D1 fluorescence microscope (Carl Zeiss, Germany). Filter sets for eGFP (Zeiss Filter Set 38; Ex/Em: 450–490/500–550 nm) and rhodamine (Zeiss Filter Set 20; Ex/Em: 540–552/567–647 nm) were used for green and red (stained bacteria NA and cyanobacteria autofluorescence) signal visualization, respectively.

### Spectrophotometric Analyses

The absorption spectra of acetone-extracted photosynthetic and light-protecting pigments (chlorophyll *a* and carotenoids) were obtained using a HP8452A Diode Array dual-beam spectrophotometer (Hewlett-Packard, Tokyo, Japan). For this purpose, the rock surface layers with its endolithic colonization zone were gently crushed in an agate mortar, and pigment extraction was performed according to Wierzchos *et al*.^6^.

### Raman spectroscopy

For point Raman measurements, a Renishaw *InVia* spectrometer (Renishaw, Wotton-under-Edge, UK) was employed with a Leica 50x magnification objective (NA/0.75). A far-red 785 nm line of a diode laser was employed with the following acquisition parameters: 3 s–5 s exposure time, 10 accumulations, and 30 mW laser power. A 514.5 nm green excitation laser wavelength was used for the resonance Raman analyses of the carotenoids. The acquisition parameters were set as follows: 1 s exposure time, 10 accumulations, and 1.25 mW laser power.

For the Raman imaging analyses, the same Renishaw *InVia* spectrometer was used, employing its streamline (linefocus) mode. A flattened transect of the ignimbrite specimen, with visually evident green colonization about 1 mm below the surface, was adjusted under the microscope to obtain a consistent focal plane. Then, the selected area was subjected to mapping. For imaging of the carotenoids, a 5x magnification objective (NA/0.12) and Ar laser line at 514.5 nm was employed with 12 mW source power, 3 s exposure time, and a spectral range of 250–2100 cm^−1^. For detailed mapping of the carotenoids, the same laser line was employed with a 20x magnification objective (NA/0.40), 2.5 mW laser power, and 4 s exposure time. A 785 nm laser line and 20x magnification objective was used for imaging of phycobiliproteins, using 150 mW laser power, and 5 s exposure time.

### Data processing

Single spectra from the point Raman analysis were baseline corrected in Grams/AI 9.1 (Thermo Scientific) or Wire 3.4 (Renishaw) using cubic spline interpolation and manual selection of the points to effectively eliminate the influence of the fluorescence background. For the purpose of comparison, spectra obtained by the 785 and 514.5 nm laser line, were normalized in Wire 3.4.

Imaging data were processed using Wire 3.4, including Matlab-based chemometric tools. First, the large datasets were truncated within the spectral range to the region of interest in order to reduce the requirements on the PC. This allowed for further processing; e.g., the region where the ν_1_ modes of carotenoids and C=C and C=N stretchings of the phycobiliproteins are expected. Then, spectral artifacts due to cosmic rays were removed using the nearest neighbor method, and the spectral noise was filtered through the datasets before map construction. The image maps, showing overall carotenoid or phycobiliprotein distributions, were created as “signal-to-baseline” intensity in the specific region of interest. For overall distribution of the carotenoids, the Raman signal in the 1485–1545 cm^−1^ region was depicted, corresponding to the ν_1_(C=C) band. For the phycobiliproteins, signal-to-baseline within the 1610–1660 cm^−1^ wavenumber range was depicted. For imaging of the distribution of carotenoid composition, the dataset was curve-fitted (based on the data previously obtained using single-point analysis), and then displayed as the “peak height” of individual curves fitted to the baseline-corrected spectral dataset. Mixed Gaussian-Lorentzian curves for two components with a band position centered at 1503 +/−2 cm^−1^, 1519 +/−2 cm^−1^ were calculated. The look-up-table (LUT) was set in order to obtain optimal contrast. Individual Raman spectra from different areas of the map were checked, and examples extracted from the imaging datasets were presented. For creation of an image combining the signals of the carotenoids and sample images, the individual layers of the images exported from Wire 3.4 software were blended in Adobe Photoshop CS6.

### Data availability

The datasets generated during and/or analyzed during the current study are available from the corresponding author on reasonable request.

## References

[1] Friedmann EI, Lipkin Y, Ocampo-Paus R (1967). Desert algae of the Negev (Israel). Phycologia.

[2] Friedmann EI, Ocampo R (1976). Endolithic blue-green-algae in Dry Valleys – primary producers in Antarctic desert ecosystem. Science.

[3] Wynn-Williams DD, Edwards HGM, Garcia-Pichel F (1999). Functional biomolecules of Antarctic stromatolitic and endolithic cyanobacterial communities. Eur. J. Phycol..

[4] Edwards HGM, Newton EM, Dickensheets DL, Wynn-Williams DD (2003). Raman spectroscopic detection of biomolecular markers from Antarctic materials: evaluation for putative Martian habitats. Spectrochim. Acta A.

[5] Wierzchos J, Ascaso C, McKay CP (2006). Endolithic cyanobacteria in halite rocks from the hyperarid core of the Atacama Desert. Astrobiology.

[6] Wierzchos J (2015). Adaptation strategies of endolithic chlorophototrophs to survive the hyperarid and extreme solar radiation environment of the Atacama Desert. Front. Microbiol..

[7] Stivaletta N, Barbieri R, Billi D (2012). Microbial colonization of the salt deposits in the driest place of the Atacama Desert (Chile). Orig. Life Evol. Biosph..

[8] Robinson CK (2015). Microbial diversity and the presence of algae in halite endolithic communities are correlated to atmospheric moisture in the hyper-arid zone of the Atacama Desert. Environ. Microbiol..

[9] Dong H, Rech JA, Jiang H, Sun H, Buck BJ (2007). Endolithic cyanobacteria in soil gypsum: occurrences in Atacama (Chile), Mojave (United States), and Al-Jafr Basin (Jordan) Deserts. J. Geophysic. Res..

[10] Wierzchos J (2011). Microbial colonization of Ca-sulfate crusts in the hyperarid core of the Atacama Desert; implications for the search for life on Mars. Geobiology.

[11] DiRuggiero J (2013). Microbial colonisation of chasmoendolithic habitats in the hyper-arid zone of the Atacama Desert. Biogeosciences.

[12] Wierzchos J (2013). Ignimbrite as a substrate for endolithic life in the hyper-arid Atacama Desert; implications for the search for life on Mars. Icarus.

[13] Anderson JC, Robertson DS (1960). Role of carotenoids in protecting chlorophyll from photodestruction. Plant Phys..

[14] Krinsky NI (1979). Carotenoid protection against oxidation. Pure Appl. Chem..

[15] Siefermann-Harms D (1987). The light-harvesting and protective functions of carotenoids in photosynthetic membranes. Physiol. Plant..

[16] Telfer, A., Pascal, A. & Gall, A. Carotenoids in Photosynthesis. In: Carotenoids (Eds: Britton, G., Liaaen-Jensen, S. & Pfander, H.), vol. 4, pp. 265–308 (Birkhäuser Basel, 2008).

[17] Adams WW, Demmig-Adams B (1992). Operation of the xanthophyll cycle in higher plants in response to diurnal changes in incident sunlight. Planta.

[18] Lunch CK (2013). The xanthophyll cycle and NPQ in diverse desert and aquatic green algae. Photosynth. Res..

[19] Holt TK, Krogmann DW (1981). A carotenoid-protein from cyanobacteria. Biochim. Biophys. Acta.

[20] Kerfeld CA (2003). The crystall structure of a cyanobacterial water-soluble carotenoid binding protein. Structure.

[21] Wilson A (2006). A soluble carotenoid protein involved in phycobilisome-related energy dissipation in cyanobacteria. Plant Cell.

[22] Rakhimberdieva MG, Stadnichuk IN, Elanskaya IV, Karapetyan NV (2004). Carotenoid-induced quenching of the phycobilisome fluorescence in photosystem II-defficient mutant of *Synechocystis* sp. FEBS Lett..

[23] Kirilovsky, D. & Kerfeld, C. A. Cyanobacterial photoprotection by the orange carotenoid protein. *Nature Plants* **2**, 16180 (2016). 10.1038/nplants.2016.180 27909300

[24] Gill D, Kilponen RG, Rimai L (1970). Resonance Raman scattering of laser radiation by vibrational modes of carotenoid pigment molecules in intact plant tissues. Nature.

[25] Merlin JC (1985). Resonance Raman spectroscopy of carotenoids and carotenoid-containing systems. Pure Appl. Chem..

[26] Wilson A (2008). A photoactive carotenoid protein acting as light intensity sensor. Proc. Nat. Acad. Sci..

[27] Withnall R, Chowdhry BZ, Silver J, Edwards HGM, de Oliveira LFC (2003). Raman spectra of carotenoids in natural products. Spectrochim. Acta A..

[28] Leverenz RL (2014). Structural and functional modularity of the Orange Carotenoid Protein: distinct roles for the N- and C-terminal domains in cyanobacterial photoprotection. Plant Cell.

[29] Leverenz RL (2015). A 12 Å carotenoid translocation in a photoswitch associated with cyanobacterial photoprotection. Science.

[30] Gupta S (2015). Local and global structural drivers for the photoactivation of the orange carotenoid protein. Proc. Nat. Acad. Sci..

[31] McKay CP (2003). Temperature and moisture conditions for life in the extreme arid region of the Atacama Desert: Four years of observations including the El Niño of 1997–1998. Astrobiology.

[32] Azúa-Bustos A, Caro-Lara L, Vicuňa R (2015). Discovery and microbial content of the driest site of the hyperarid Atacama Desert, Chile. Environ. Microbiol. Rep..

[33] Cámara B (2015). Ignimbrite textural properties as determinants of endolithic colonization patterns from hyper-arid Atacama Desert. Int. Microbiol..

[34] Crits-Christoph A (2016). Phylogenetic and Functional Substrate Specificity for Endolithic Microbial Communities in Hyper-Arid Environments. Front. Microbiol..

[35] Rondanelli R, Molina A, Falvey M (2015). The Atacama surface solar maximum. Bull. Am. Meteorol. Soc..

[36] Cabrol NA (2014). Record solar UV irradiance in the tropical Andes. Front. Environ. Sci..

[37] Vítek P (2013). Phototrophic community in gypsum crust from the Atacama Desert studied by Raman spectroscopy and microscopic imaging. Geomicrobiol. J..

[38] Szalontai B, Gombos Z, Csizmadia V, Bagyinka C, Lutz M (1994). Structure and interactions of phycocyanobilin chromophores in phycocyanin and allophycocyanin from an analysis of their resonance Raman spectra. Biochemistry.

[39] de Oliveira VE, Castro HS, Edwards HGM, de Oliveira LFC (2010). Carotenes and carotenoids in natural biological samples: a Raman spectroscopic analysis. J. Raman Spectrosc..

[40] Liaaen-Jensen S (1997). Stereochemical aspects of carotenoids. Pure Appl. Chem..

[41] Vítek P, Ascaso C, Artieda O, Wierzchos J (2016). Raman imaging in geomicrobiology: endolithic phototrophic microorganisms in gypsum from the extreme sun irradiation area in the Atacama Desert. Anal. Bioanal. Chem..

[42] Villar SEJ, Edwards HGM, Benning LG (2006). Raman spectroscopic and scanning electron microscopic analysis of a novel biological colonisation of volcanic rocks. Icarus.

[43] Oren A, Kühl M, Karsten U (1995). An endoevaporitic microbial mat within a gypsum crust: zonation of phototrophs, photopigments, and light penetration. Mar. Ecol.-Progr. Ser..

[44] Culka A (2014). Detection of pigments of halophilic endoliths from gypsum: Raman portable instrument and European Space Agency’s prototype analysis. Philos. Trans. Roy. Soc. A.

[45] Saito T, Miyabe Y, Ide H, Yamamoto O (1997). Hydroxyl radical scavenging ability of bacterioruberin. Radiat. Phys. Chem..

[46] Punginelli C, Wilson A, Routaboul J-M, Kirilovsky D (2009). Influence of zeaxanthin and echinenone binding on the activity of the Orange Carotenoid Protein. Biochim. Biophys. Acta.

[47] Jallet D (2014). Specificity of the cyanobacterial Orange Carotenoid Protein: Influences of Orange Carotenoid Protein and Phycobilisome Structures. Plant Physiol..

[48] Kirilovsky D, Kerfeld CA (2013). The Orange Carotenoid Protein: a blue-green light photoactive protein. Photochem. Photobiol. Sci..

[49] Krieger-Liszkay A, Fufezan C, Trebst A (2008). Singlet oxygen production in photosystem II and related protection mechanism. Photosynt. Res..

[50] Boulay C, Abasova L, Six C, Vass I, Kirilovsky D (2008). Occurrence and function of the orange carotenoid protein in photoprotective mechanisms in various cyanobacteria. Biochim. Biophys. Acta.

[51] Maksimov EG (2015). The signaling state of orange carotenoid protein. Biophys. J..

[52] Koyama Y, Takatsuka I, Nakata M, Tasumi M (1988). Raman and infrared spectra of the all-trans, 7-cis, 9-cis, 13-cis and 15-cis isomers of β-carotene: Key bands distinguishing stretched or terminal-bent configurations form central-bent configurations. J. Raman Spectrosc..

[53] González G, Cembrano J, Aron F, Veloso EE, Shyu JBH (2009). Coeval compressional deformation and volcanism in the central Andes, case studies from northern Chile (23°S–24°S). Tectonics.

